# Supplementary material for: Sudden cardiac death after coronary artery bypass graft surgery and role of antiplatelet therapy
Source: PLoS One. 2025 Feb 25;20(2):e0319292. doi: 10.1371/journal.pone.0319292 (PMC11856524; doi:10.1371/journal.pone.0319292)
Supplement: Attachment — Submitted filename: PLOS Rebuttal Letter to the Editor and Reviewers.docx [file pone.0319292.s001.docx]

**Rebuttal Letter to the Editor and Reviewers – PLOS ONE Journal**

Dear Editor and Reviewers,

We sincerely thank you for your detailed and constructive feedback, which has been invaluable in improving the clarity and rigor of our manuscript. Below, we address each reviewer’s comments and outline the revisions made. We did not identify any retracted studies among the cited references at this time. However, please let us know if we have inadvertently overlooked any retracted references.

**Reviewer 1**

**Q1:**
*I’d suggest magnifying the tables and graphics references in the results part of the manuscript, permitting the reader to rapidly identify the references.*

**Response:**
Thank you for this valuable suggestion. We have revised the manuscript to ensure that references to tables and graphics in the results section are now more prominent and easily identifiable.

**Reviewer 2**

**Q2:**
*What kinds of sudden cardiac death (SCD) are reduced by DAPT, and what is the mechanism? In comparison to aspirin monotherapy (AMT), the main benefit of DAPT can possibly reduce acute major adverse cardiac and cerebrovascular events (MACCE), defined as a composite of death, myocardial infarction, stroke, or repeat revascularization, but not death caused by heart failure and arrhythmia. In a previous propensity-matched analysis, DAPT did not confer any advantage in terms of improved survival or freedom from MACCE compared to aspirin monotherapy following isolated CABG.*

**Response:**
We appreciate the reviewer’s insightful comments. DAPT appears to reduce early mortality within the first 3 months post-CABG by mitigating thrombotic graft occlusion, a critical mechanism underlying the observed survival benefit. Unlike the referenced study, which relied solely on discharge records and didn’t ensure compliance, we manually reviewed patient charts and confirmed antiplatelet prescription compliance during follow-ups. Additionally, we employed stricter and clinically relevant exclusion criteria, excluding only deaths within 48 hours post-surgery, whereas the referenced study excluded all in-hospital deaths, potentially underestimating DAPT's benefits. While Hess et al. did not demonstrate significant MACCE benefits, their data showed a trend toward better survival with DAPT. Current ACC/AHA guidelines support DAPT post-CABG in ACS patients, aligning with our findings of improved early postoperative survival. We have clarified this in the revised manuscript. Additionally in our discussion we have provided citations to several studies proving survival benefit of DAPT after CABG including study by Sorenson et al and various other studies (refer to citations 10-14) have shown promising results with DAPT treatments.

Reference: *Sørensen R, Abildstrøm SZ, Hansen PR, Hvelplund A, Andersson C, Charlot M, Fosbøl EL, Køber L, Madsen JK, Gislason GH, Torp-Pedersen C. Efficacy of post-operative clopidogrel treatment in patients revascularized with coronary artery bypass grafting after myocardial infarction. Journal of the American College of Cardiology. 2011 Mar 8;57(10):1202-9.*

**Q3:**
*In this study, the authors didn’t demonstrate the difference in post-CABG acute coronary syndrome (ACS) and myocardial infarction (MI) between the AMT and DAPT groups, which are possible main reasons for reducing SCD by DAPT and P2Y12 antagonists.*

**Response:**
We agree with the reviewer that sudden cardiac deaths due to VT/VF or unwitnessed SCD are likely attributable to fatal MI and/or ischemic events. However, in our study, no significant differences were observed between the AMT and DAPT groups in the incidence of non-fatal MI, CVA, or the composite of these events as MACE.

**Q4:**
*The level of low-density lipoprotein (LDL) and the significant proportion of statin and anti-arrhythmic drug (AAD) administration are also important confounding factors for SCD. I suggest listing these and performing univariate and multivariate Cox regression analysis.*

**Response:**
We conducted multivariate analysis and found that neither statins nor AADs showed significant protective effects warranting inclusion in the final hazard ratios. We focused on clinically meaningful covariates in the analysis. Unfortunately, DAPT appears to reduce early mortality within the first 3 months post-CABG by preventing thrombotic graft occlusion, a key mechanism underlying the observed survival benefit and the primary objective of this treatment strategy

We have added the following statement to the manuscript:
“Common post-CABG medications, including ACE inhibitors/ARBs, beta-blockers, antiarrhythmic drugs (AAD), calcium channel blockers (CCBs), and statins, were not significant predictors of SCD (p=0.380, 0.450, 0.428, 0.337, and 0.185, respectively).”
The role of statins in improving overall survival (but not SCD) post-CABG is addressed in a separate article under review.

Also we have added a statement under limitations, “Another limitation of this study is that, apart from antiplatelet therapy, which was carefully verified for both type and duration of treatment, most other medications, such as statins and beta blockers, were recorded based on the discharge medication list at the time of surgery. Consequently, there may be cases where patients initially prescribed lower-intensity statins were later switched to high-intensity therapy, or where patients were not prescribed statins or had their statin therapy discontinued due to intolerance.”

**Limitations**

**Reviewer 1:**
*Suggested limitations have been added.*

**Response:**
We appreciate the reviewer’s comments and have incorporated the suggested limitations into the revised manuscript. These include the retrospective nature of the study, single-center experience, and potential bias in correctly identifying SCD in the AMT group. Additionally, the absence of propensity matching between the AMT and DAPT groups has been noted as a limitation.

Thank you for your constructive feedback, which has strengthened our manuscript.

Sincerely,
Iftikhar Ali Ch, MD

South Oklahoma Research

5200 E Interstate 240 Service Road, Oklahoma City, OK 73135

Tel: 405-628-6000 | Fax Number: 405-628-0000 | E-mail: babar_175@hotmail.com
